# Development of Multicellular Tumor Spheroid (MCTS) Culture from Breast Cancer Cell and a High Throughput Screening Method Using the MTT Assay

**DOI:** 10.1371/journal.pone.0044640

**Published:** 2012-09-06

**Authors:** Wan Yong Ho, Swee Keong Yeap, Chai Ling Ho, Raha Abdul Rahim, Noorjahan Banu Alitheen

**Affiliations:** 1 Department of Cell and Molecular Biology, Faculty of Biotechnology and Biomolecular Sciences, Universiti Putra Malaysia, Serdang, Selangor, Malaysia; 2 Institute of Biosciences, Universiti Putra Malaysia, Serdang, Selangor, Malaysia; University of Texas MD Anderson Cancer Center, United States of America

## Abstract

In comparison to monolayer cells, MCTS has been claimed as more suitable candidate for studying drug penetration due to the high resemblance to solid tumors. However, the cultivation of MCTS is cumbersome, time consuming, and most technique fail to generate spheroids with uniform sizes. Therefore, the application of spheroid cultures in high throughput screening has been rather limiting. Besides, the lack of a well established screening protocol method that is applicable to spheroid could also be attributed to this limitation. Here we report a simple way of cultivating homogenous MCTS cultures with compact and rigid structure from the MCF-7 cells. Besides, we had also made some modifications to the standard MTT assay to realize high throughput screening of these spheroids. Using the modified protocol, tamoxifen showed cytotoxicity effect towards MCTS cultures from MCF-7 with high consistency. The results correlated well with the cultures’ response assessed by LDH release assay but the latter assay was not ideal for detecting a wide range of cytotoxicity due to high basal background reading. The MTT assay emerged as a better indicator to apoptosis event in comparison to the LDH release assay. Therefore, the method for spheroid generation and the modified MTT assay we reported here could be potentially applied to high throughput screening for response of spheroid cultures generated from MCF-7 as well as other cancer cell lines towards cytotoxic stimuli.

## Introduction

Monolayer cultures have been used extensively in cancer research for studies involving the regulation of cell growth and cell death [1]. However, monolayer cultures are more susceptible to the cytotoxic insult by xenobiotics in comparison to tumors *in vivo* due to their lack of microenvironmental properties and cellular activities that take place in solid tumors [2]. Therefore, the three-dimensional multicellular tumor spheroidal (MCTS) culture has been proposed as a valuable model to provide more comprehensive assessment of tumor in response to therapeutic strategies [3].

MCTS was defined by Hamilton (1998) as ‘spherically symmetric aggregates of cells analogous to tissues, with no artificial substrate for cell attachment’. It mimics tumors *in vivo* in many ways, such as the expression of antigens, pH and oxygen gradients within its microenvironment, penetration rate of growth factors and distribution of proliferating/quiescent cells within the spheroid [3]. Not only does the arrangement of cells in a three dimensional organization differ to that in the monolayer form, the growth pattern and protein expression of spheroid [4], as well as its interaction with extracellular matrix [5] were also found to resemble those of the solid tumors compared to monolayer cultures. At such, the accessibility of cytotoxic agents into the spheroids may be limited by hypoxia and poor vascularisation within the microregions of the cultures [6] as occur in solid tumors [7]. This further demonstrate that spheroids are more suitable models for drug penetration studies in tumors in comparison to monolayer cells [3].

However, the application of MCTS for high-throughput screening is limited due to long cultivation time, cumbersome culturing technique, formation of unequal-size spheroid and failure to produce rigid aggregates [8]. Spheroid cultures of homogenous sizes and growth characteristic are important factors that greatly affect the precise quantification of biological or biochemical endpoints in drug screening [9]. Furthermore, the lack of a simple and well-established procedure for rapid generation of MCTS cultures may be another reason for the limited use of this three-dimensional culture system in drug screening process [10].

The 3-(4,5-dimethylthiazol-2-yl)-2,5-diphenyl tetrazolium bromide (MTT) assay is one of the most widely used methods for cytotoxicity screening due to its simple and rapid procedure [11]. MTT is a tetrazolium salt that can be cleaved only by active mitochondria in metabolically active cells, and is hence applicable to almost any survival or proliferation assay in which living cells must be distinguished from the dead ones [12]. The assay, which could be carried out in multiwell plates, also offers an advantage for testing a large number of drugs with good reproducibility [13]. However, the use of MTT assay for drug screening on MCTS cultures is rare. A possible reason for this could be due to the lack of a standardized technique to incorporate the use of the MTT assay into studies involving MCTS.

Therefore, the present study was carried out to develop a stable, homogenous and reproducible MCTS culture from MCF-7 and to modify the standard procedures of the MTT assay to enable its application for high throughput screening of anticancer drug involving MCTS cultures. The method was then compared to the LDH release assay, one of the most common approaches for cytotoxicity testing of cells grown as spheroids [14], [15], [16]. To validate the results from both assays, a flow cytometric analysis of phosphatidyl externalization was also carried out.

**Figure 1:**
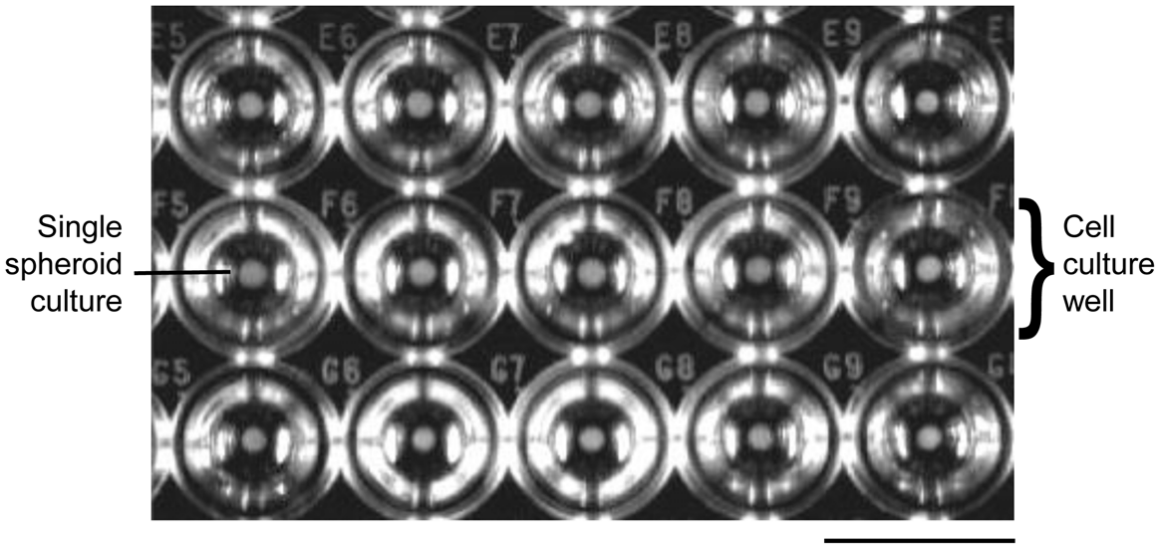
Muticellular tumor spheroidal culture of MCF-7 with homogenous sizes. The cells were cultured in a 96-well plate that was coated with agar gelrite. Cell aggregation was facilitated by centrifugation without the addition of any extracellular matrix to induce the formation of spheroid (Magnification: 1x, scale bar: 1 cm).

**Figure 2:**
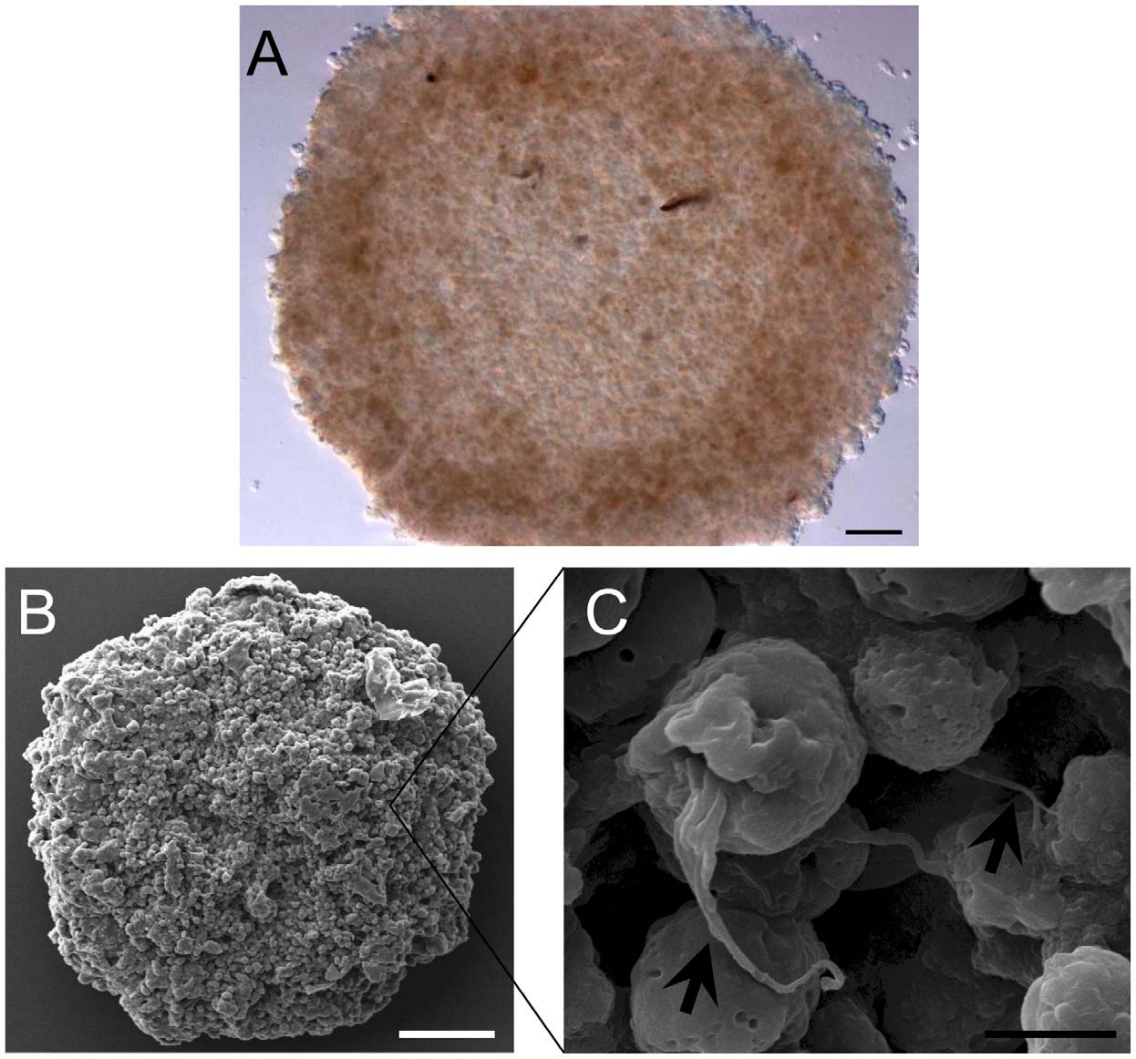
Morphological appearance of MCTS culture of MCF-7. (A) With the facilitation by centrifugal force, MCF-7 cells were organized into a three-dimensional multicellular spheroidal structure. Under phase contrast microscope, the structure appeared to be compact and the cells were rigidly integrated into the solid structure where individual cells were indistinguishable from each other (magnification: 40x, scale bar: 100 µm). (B) Appearance of the culture under scanning electron microscopy (magnification: 160x, scale bar: 100 µm). (C) Single cells within the spheroid culture were connected to adjacent cells through cell-cell junction (arrow), which were responsible for the densely packed organization of the cells (magnification: 4000x, scale bar: 5 µm).

## Materials and Methods

### Cell Culture

The estrogen-dependent human breast adenocarcinoma, MCF-7 was obtained from American Type Culture Collection (ATCC, USA). The cells were maintained in Dulbecco’s modified eagle medium (DMEM) (Sigma, USA) supplemented with 10% (v/v) of heat-inactivated foetal bovine serum (FBS) (PAA, Austria), 100 I.U./ml penicillin and 100 ng/ml streptomycin (PAA, Austria). The cells were cultured at 37°C in a 90% humidified incubator with 5% CO_2_.

### Generation and Treatment of Multicellular Tumor Spheroidal (MCTS) Cultures from MCF-7

The generation of spheroids was carried out using a liquid overlay cultivation technique. To produce a non-adherent condition for the development of MCTS culture, a 96-well plate was pre-coated with 1% (w/v) agar. Then, single cell suspension of MCF-7 at a density of 5×10^4^ cells in 200 μl of DMEM was loaded into each well. Aggregation of cells was facilitated by centrifugation of the plate at 1,000 × g for 5 minutes. Then, the plate was incubated at 37°C in a 90% humidified incubator with 5% CO_2_ for 3 days.

For treatment, the 3 day-old spheroids were transferred to a new 96 well plate that was pre-coated with agar. Each well was added with 200 μl of freshly prepared medium containing tamoxifen with concentrations ranged between 0–90 μM. The cultures were then incubated for 4 days prior to MTT, LDH release and flow cytometric apoptosis assays.

### Light Microscopic Assessment

Morphological appearances of the MCTS cultures and their structural changes after 4 days of treatment by tamoxifen were observed using a Nikon Diaphot-TMD (Nikon, Japan) inverted light microscope equipped with Phase contrast-2 ELWD 0.3 phase-contrast condenser and the images were captured using the Digital sight DS-L2 camera (Nikon, Japan).

**Figure 3:**
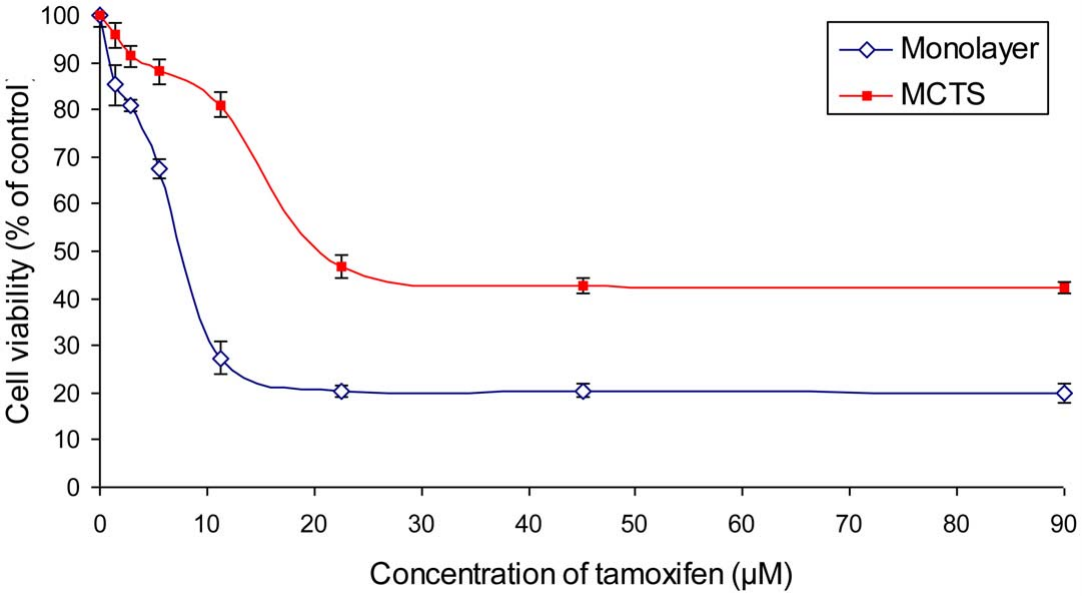
Viability of the monolayer and MCTS cultures of MCF-7 determined from the MTT assay. Monolayer and MCTS cultures of the MCF-7 cell line were exposed to tamoxifen for 4 days. MTT assay for the MCTS cultures was carried out with some modification to the standard protocol by Mosmann (1983) [11] while assay for the monolayer culture was performed in accordance to the original method.

### Scanning Electron Microscopic Observation of Spheroid Culture

MCTS cultures were incubated for 4 days with or without treatment by tamoxifen. Then, the cultures were washed twice with 1 ml of PBS-BSA-EDTA, followed by overnight fixing with 2% glutaraldehyde in PBS at room temperature. After that, washing with PBS-BSA-EDTA was repeated twice for 10 minutes each. The washed cultures were then dehydrated in ethanol at increasing concentrations (35%, 50%, 75% and 95%) for 10 minutes each, followed by a final dehydration in 3 changes of 100% ethanol for 10 minutes each. Subsequently, the samples were dried in a Polaron CPD 7501 critical point dryer (Polaron, UK) for 1 hour by using liquid CO_2_ as a transition medium. After mounting, the samples were subjected to gold sputter coating in SC500 Sputter Coater (Bio-Rad Laboratories, Inc. USA). Finally, the samples were examined under a LEO 1450VP scanning electron microscope (LEO, Germany) at 15 kV.

### MTT Cell Viability Assay

MTT assay for the monolayer culture was carried out according to the method by Mosmann (1983) [11] while the assay for the MCTS cultures was carried out with slight modification to the standard protocol. After 4 days of treatment, 20µl of MTT solution was added into each well containing the monolayer or MCTS cultures and incubated for 4 hours. While the monolayer culture was left untouched in the original plate, the content of each well containing the MCTS culture was transferred to a new, flat-bottom 96-well plate before the plate was centrifuged at 1,000 × g for 5 minutes. Then, 150 µl of media was aspirated from each well from the plates containing the monolayer and MCTS cultures. The plates were then blot dried on paper towels, followed by the addition of 100 µl of DMSO. Finally, absorbance was recorded at 570 nm using the µQuant enzyme-linked immunosorbent assay (ELISA) Reader (Bio-tek Instruments, USA). The concentrations of tamoxifen that resulted in 50% of cell death (IC_50_) in both the monolayer and spheroid cultures were determined from respective dose–response curves. The assay was carried out with 12 replicates for each culture.

**Figure 4:**
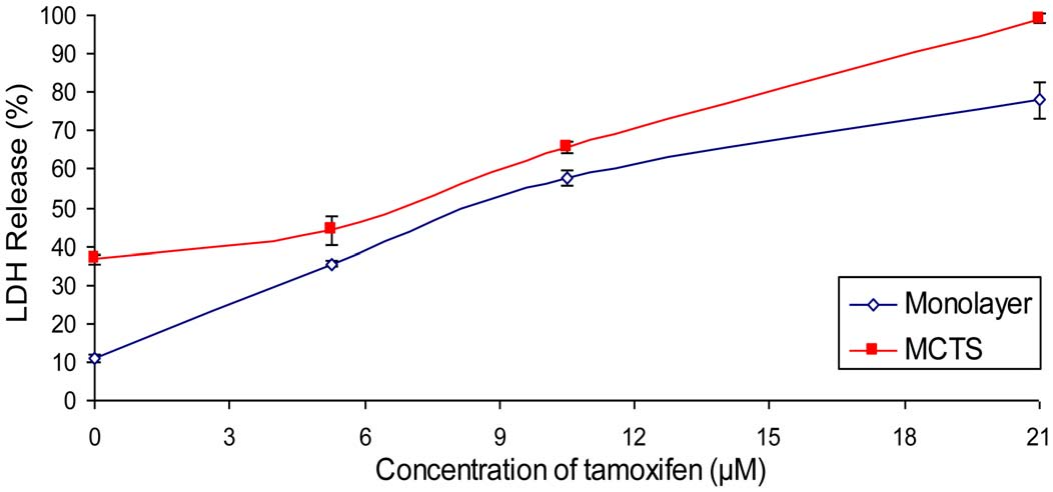
Cytotoxicity of tamoxifen citrate against monolayer and MCTS cultures of MCF-7. Cytotoxicity was determined by quantifying the percentage of LDH release relative to the maximum LDH release of untreated spheroid cultures. The results were presented as means ± S.E.M. of three independent experiments.

### LDH Cytotoxicity Assay

The cytotoxicity of tamoxifen against the monolayer and MCTS cultures of MCF-7 was assessed using the CytoTox 96® Non-Radioactive cytotoxicity assay kit (Promega, USA) according to the manufacturer’s instructions. The release of LDH into the culture medium was measured. First, 20 μl of 10 x lysis solution was added into wells containing the untreated control cells (for both monolayer and MCTS cultures) prior to the assay to induce maximum LDH release. After 45 minutes, the plate was centrifuged at 300 ×g for 4 minutes and 50 µl of the supernatant from each well (including untreated control, treated and maximum LDH release groups) was transferred to a new plate. Subsequently, 50 μl of TMB coupled enzymatic substrate buffer was added into each well and incubated at room temperature for 30 minutes in the dark. Stop solution (50 μl) was added into each well and the absorbance was measured at 492 nm using the µ Quant ELISA Reader (Bio-tek Instruments, USA). The percentage of cytotoxicity was calculated according to the following formula:
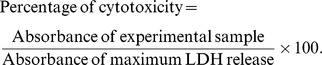



**Figure 5:**
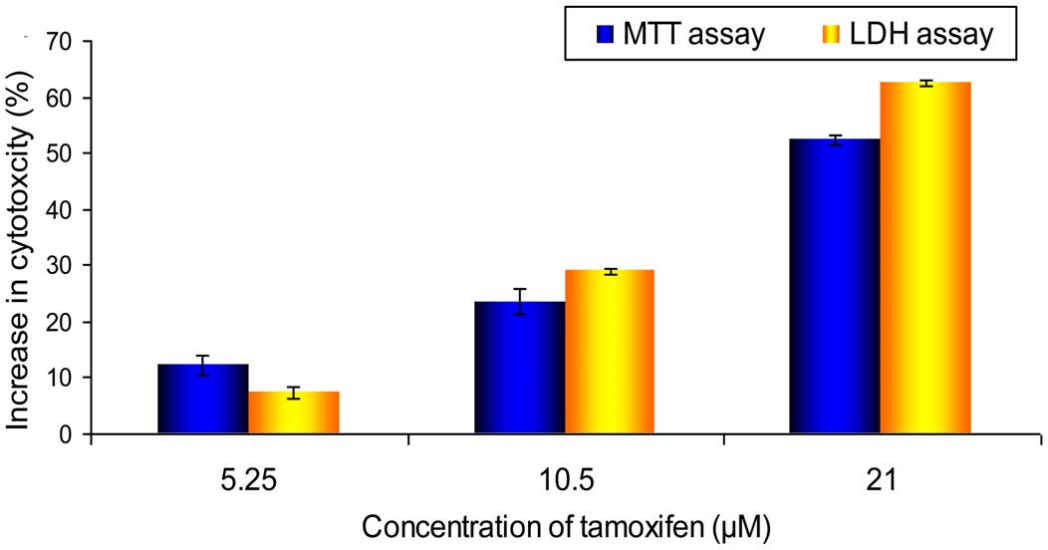
Comparison of the cytotoxicity increment in MCTS cultures assessed by MTT and LDH release assays. Relative to the untreated control, which was normalized to 0% in both assays, reduction in cell viability of treated samples assessed by MTT assay was compared to the elevation in cytotoxicity determined from the LDH release assay. The results were presented as means ± S.E.M. of three independent experiments.

### Comparison between Results from the MTT and LDH Assays for MCTS Culture

The basal LDH leakage in the control MCTS culture was eliminated to compare the results obtained from the MTT assay and the LDH release assay for MCTS culture. At such, the % of cytotoxicity of the untreated control was normalized to 0% and the elevation in LDH release by each concentration of tamoxifen was calculated by subtracting the basal value of LSH release (36.59) from their respective % of LDH release as follows:

Elevation in LDH release  =  % of LDH release by treatment group − % of LDH release by untreated control (36.59%)

By assuming that the reduction in cell viability in the MTT assay was proportional to the increment in cytotoxicity, we made a pairwise comparison of the increase in cytotoxicity by different concentrations of tamoxifen for both assays.

### Flow Cytometric Detection of Apoptosis by Annexin V/PI Staining

Before the analysis, spheroid cultures were disaggregated into single cell suspension using enzymatic dissociation. Briefly, the cultures were washed twice in 1 ml of PBS-BSA-EDTA solution followed by incubation in 500 μl of Accutase™ solution (PAA, Austria) at 37°C for 20 minutes to allow complete dissociation of cells. The cells were then centrifuged at 300 ×g for 5 minutes followed by washing twice with PBS-BSA-EDTA solution. Phosphatidylserine (PS) externalization was assessed by quantifying surface annexin V-FITC and propidium iodide (PI) (Becton Dickinson, USA) using the FACS Calibur flow cytometer (Becton Dickinson, USA). The staining and analysis were performed as previously described [17].

### Statistical Analysis

Statistical analysis was performed using SPSS 13.0 software (SPSS Inc., USA). Results were expressed as mean ± standard error (S.E.M.). Differences between means were evaluated using one-way analysis of ANOVA followed by Duncan test. A p value of ≤0.05 was taken as statistically significant.

## Results

### Generation of MCTS Cultures with Homogenous Size for Cytotoxicity Evaluation

The generation of MCTS cultures with homogenous sizes was essential for cytotoxic screening. The coating of agar gelrite not only produced a non-adherent environment to avoid attachment of the cells to cell culture plate but also prepared a concave surface for the formation of cells into spheroidal shape. Centrifugation facilitated the formation of these spheres by pooling all the cells together from an early stage of aggregation. During the first 3 days of incubation, the cells started to aggregate but the aggregates could be easily dissociated by mechanical force. However, incubation for 4 days alone was sufficient for these cells to form a compact and rigid sphere without the aid of any basement membrane matrix. The “matured” spheroids that were formed were uniform with a diameter of about 1.1±0.1 mm and a cell number of about 5.12±0.12×10^4^ cells each (Figure 1). When observed under phase contrast microscope, the cultures appeared as a multilayer cell assembly that was densely packed at the central core region (Figure 2A). Under scanning electron microscope at 160x magnification (Figure 2B), thousands of cells were shown to interconnect with one another in the form of a three-dimensional spherical aggregate and distinct cells were indistinguishable. At a higher magnification (4000×), these cells were shown to be held together at the cell-cell junctions throughout the whole MCTS culture (Figure 2C). Besides, the cultures were also resistant to gentle agitation or physical transfer and only enzyme dissociation could separate these cultures into single cells.

### Tamoxifen Induced Cell Death in both Monolayer Culture and Spheroids of MCF-7

We have made some modifications to the standard MTT assay by Mosmann [11] to determine the viability of the spheroids. Before the addition of MTT solution, the content of all the wells were transferred to a new, non-coated plate to eliminate interference to the absorbance reading by the coated agar in the original plate. After incubation with MTT, the plate was centrifuged to sediment the solid content of all wells. However, only 150 µl of media was aspirated to avoid accidental removal of the blue formazan formed. Removing the remaining liquid by inverting and blotting the plate onto paper towels was found to be more effective in retaining formazan crystals in the wells. However, we did not blot the plate directly onto paper towels prior to the aspiration of media as this would cause some crystals to leak out during the process.

**Table 1:** Induction of apoptosis in MCTS cultures of MCF-7 after 4-day exposure to different concentrations of tamoxifen.

	Untreated control	Tamoxifen concentration (µM)		
		5.25	10.5	21
Viable	87.90±0.55	57.50±6.37*	42.06±5.02*	30.34±0.83*
Early apoptosis	0.13±0.04	10.26±1.27*	20.16±4.93*	40.07±0.64*
Late apoptosis	0.01±0.01	25.71±1.14*	31.33±0.25*	24.02±1.82*
Necrosis	11.95±0.51	6.53±2.77*	6.47±0.15*	5.59±1.62*

* Statistical significance (P<0.05) between control cells and treatment groups.

From a serial dilution of tamoxifen (90, 45, 22.5, 11.25, 5.63, 2.81 and 1.41 µM), the IC_50_ value obtained for the monolayer culture was 7.74±0.19 µM while the IC_50_ value for MCTS was 20.62±1.66 µM (Figure 3). From the regression curves, we observed a dosage dependent cytotoxicity induction by tamoxifen in both cultures. However, a plateau phase or perhaps maximum cell killing effect was reached for both cultures at concentrations higher than 22.5 µM. Although disparity in cell viability between the cultures persisted at all concentrations, both cultures exhibited a similar pattern of response curve. This implied to us that having the MCF-7 cells to grow in the form of spheroid did not change the dosage dependent cytotoxicity of the drug. However, MCTS was shown to confer a higher resistance to tamoxifen treatment in comparison to its monolayer counterpart. This could be attributed to the cytotoxic barrier generated by the arrangement of the MCF-7 cells into multilayer, which was termed “multicellular resistance”. On the other hand, these results also suggested that the MTT assay was not only suitable for assessing cell viability in monolayer culture but is also reliable for viability assessment involving the MCTS cultures.

### Lactate Dehydrogenase (LDH) Release by MCTS Cultures of MCF-7 Treated with Tamoxifen

The release of LDH from cells has been used in short-term cytotoxicity assays to provide a sensitive and quantitative measure of cytotoxicity [18]. Without having to detach monolayer culture from the multi-well plates or disrupt the integrity of the spheroids, the quantification of LDH release into extracellular medium could be determined. Therefore, the effect of tamoxifen on MCTS could be examined without prior dissociation of the cultures. As shown in Figure 4, the basal amount of LDH release by the untreated monolayer culture was 10.90±0.88% while by the untreated MCTS cultures was 36.59±1.31% of the maximum LDH content (Figure 4). As the concentration of tamoxifen was elevated to 5.25, 10.5 and 21 µM, the release of LDH also increased in a concentration dependent manner in both types of cultures.

Similar to the findings from the MTT assay, we noticed again that the trend of LDH release for both types of cultures was rather similar. However, the amount of LDH release was higher for all MCTS groups in comparison to their monolayer counterparts. The high basal value of LDH in the control MCTS cultures could be related to the presence of necrotic cores in the inner part of the spheroid cultures [1], [6], [19]. This central region is highly susceptible to cell death and necrosis due to oxygen and nutritional deprivation. Depending on the size of a spheroid, the necrotic region may account for 35 to 55% of the mass volume of the culture [20]. As the occurrence of necrosis is highly associated to cell membrane leakage [21], this could potentially induce a higher amount of LDH leakage into the extracellular medium.

### Comparison between Results from the MTT and LDH Assays

From Figure 5, the differences in % of cytotoxicity were calculated based on normalization of the cytotoxicity of control group from the LDH assay to 0% to allow direct comparison of the results with that of the MTT assay. The results showed that the reduction of cell viability in the MTT assay correlated well with increase of LDH release in the MCTS culture.

### Apoptosis of Spheroid in Response to Tamoxifen Exposure

Complete dissociation of the cells without affecting their viability was crucial for precise detection of apoptosis of MCTS cultures using flow cytometry. In this assay, the spheroids were dissociated by using enzymatic reaction and the viability of individual cells was preserved after dissociation. Staining with Annexin V-FITC and PI solution allowed us to differentiate between early apoptotic and late apoptotic cell population [22]. Exposure of tamoxifen at increasing concentrations resulted in declining viability of the cultures (Table 1). The population of cells in the late apoptotic phase increased significantly in comparison to the negative control and was more prominent after treatment by 10.5 µM of tamoxifen. On the other hand, the population of early apoptotic cells increased by two fold when the concentration of tamoxifen doubled.

## Discussion

The advantage of using MCTS over monolayer culture in drug screening has been reviewed extensively [4], [5], [7], [20], [23], [24]. However, a high throughput screening that involves the use of MCTS cultures is limiting due to the difficulty to obtain homogenous spheroid cultures [8]. Nevertheless, the susceptibility of these cultures to disaggregation during treatment posed another constraint for using spheroids in high-throughput screening. To address the need for producing spheroids with uniform sizes, we cultured the MCF-7 cells using 96-well plates (Figure 1) to generate homogenous cultures that were highly reproducible. The cultures were compact, rigid and have high tolerance to mechanical force thus enabling the cultures to be transferred to a new medium for drug treatment without affecting the physical characteristics of the cultures. Besides, culturing the spheroids in multiwell plates also provided the ease of analyzing each spheroid as individual replicate, and hence eliminating the need for pooling a large amount of spheroids for cytotoxic assays.

However, the application of spheroids in high throughput screening still remains as a major challenge in anti-cancer studies. Apart from the need for rapid generation of spheroids, a well-established screening procedure that is applicable to spheroids is another prerequisite in persisting MCTS application for high throughput cytotoxic screening. In this study, MTT assay, a common method for *in vitro* screening [25], was chosen as the approach for the screening of the spheroids. The content of the wells were transferred to a new plate before the addition of MTT as a way to remove the coated agar, but this did not affect the accuracy and reproducibility of the viability measurement (Figure 3).

The reduction of viability of MCTS cultures in the MTT assay (Figure 3) was not proportionate to the increase in LDH release (Figure 4) although the differences between the control and treated groups of both assays were shown to correlate well with each other in Figure 5. This was caused mainly by the high basal release of LDH by the MCTS cultures. Hence, the application of LDH release assay may not be suitable to measure cytotoxicity in MCTS cultures which have a substantial number of necrotic cells.

To validate the results, we have compared the amount of cell death from both assays with the results from flow cytometry. Although the treatment with 21 µM of tamoxifen was shown to release approximately 99.23% of LDH of the maximum LDH control (Figure 4), it only caused about 52.33% of cell death in the MTT assay (Figure 3) and 69.67% of apoptosis and necrosis in the Annexin V/PI assay (Table 1). It was apparent from both the MTT and Annexin V/PI assays that exposure to 21 µM of tamoxifen did not give rise to maximal cell death of the spheroids. Again, the high basal degree of LDH leakage would be responsible for the remarkably high percentage of cytotoxicity in comparison to the other two methods. It could therefore be difficult to determine cytotoxicity of cultures that achieved cell death of more than 50% in the MTT assay using the LDH measurement.

Various approaches are available for the determination of cytotoxicity, but the selection of a more suitable method is important for determining the outcome of a screening. In the present study, the MTT assay was compared to LDH release assay for the practicality in assessing the cytotoxicity of drug in spheroid cultures. Although both assays show increased toxicity or cell death with increasing concentrations of drug, MTT assay was shown to be a more suitable method for assessing the effect of cells in response to drug exposure. The large scale screening of spheroids is feasible as the assay is carried out in multiwell plates. This method is not only simple, rapid and precise, but also offers good reproducibility [11]. Besides, the cost of MTT assay is also relatively cheap. It would be economic to choose this method as the preliminary screening method before more comprehensive and costly analyses are performed.

In this paper, we have introduced a simple and rapid procedure for producing uniform spheroids that were suitable for *in vitro* assays. Appealing to the need for preventing disaggregation during physical transfer, these spheroids are rigid and compact, making them a reliable tool in drug screening. Moreover, we also demonstrated the MTT assay as a suitable method for high throughput screening of cytotoxicity involving the MCTS cultures through slight modification to the standard method. Besides MCF-7, the practicability of these techniques had also been validated on other cell lines such as Hep G2, HeLa and Vero (results not shown). Therefore, the methods could also be benefited for high throughput screening of spheroids generated from other cell lines.
